# Atomic-Scale Characterization of Droplet Epitaxy Quantum Dots

**DOI:** 10.3390/nano11010085

**Published:** 2021-01-03

**Authors:** Raja S. R. Gajjela, Paul M. Koenraad

**Affiliations:** Department of Applied Physics, Eindhoven University of Technology, 5612 AZ Eindhoven, The Netherlands; P.M.Koenraad@tue.nl

**Keywords:** quantum dots, droplet epitaxy, cross-sectional scanning tunneling microscopy, atom probe tomography, optoelectronics

## Abstract

The fundamental understanding of quantum dot (QD) growth mechanism is essential to improve QD based optoelectronic devices. The size, shape, composition, and density of the QDs strongly influence the optoelectronic properties of the QDs. In this article, we present a detailed review on atomic-scale characterization of droplet epitaxy quantum dots by cross-sectional scanning tunneling microscopy (X-STM) and atom probe tomography (APT). We will discuss both strain-free GaAs/AlGaAs QDs and strained InAs/InP QDs grown by droplet epitaxy. The effects of various growth conditions on morphology and composition are presented. The efficiency of methods such as flushing technique is shown by comparing with conventional droplet epitaxy QDs to further gain control over QD height. A detailed characterization of etch pits in both QD systems is provided by X-STM and APT. This review presents an overview of detailed structural and compositional analysis that have assisted in improving the fabrication of QD based optoelectronic devices grown by droplet epitaxy.

## 1. Introduction

Semiconductor quantum dots (QDs) have been extensively studied in the last few decades due to their unique optoelectronic properties. Semiconductor QDs are nanostructures that can confine charge carriers in three spatial dimensions providing a set of discrete energy levels. Quantum confinement effects dominates when the size of at least one spatial dimension of the confining region is smaller than the de Broglie’s wavelength. The optoelectronic properties of QDs are therefore strongly influenced by the size, shape, and composition of the QDs. Precise tuning of the geometry and composition of QDs allows the optimization of both optical and electronic properties [1]. Epitaxially grown III-V semiconductor QDs offer such precise control where it has been shown that droplet epitaxy (DE) allows for a larger freedom in tuning the structural properties of quantum dot properties than is possible for quantum dots that are formed via the more common strain induced Stranski-Krastanov (SK) growth mode. By careful band gap engineering has it been possible to optimize QD nanostructures for various applications such as lasers [2,3,4,5,6,7], single and entangled photon emitters [8,9,10,11,12,13,14], photovoltaics [15,16,17,18], quantum dot infrared photodetectors [19,20,21,22] etc. Furthermore, the QDs are considered as promising building blocks for various quantum technologies such as quantum computing, quantum communication and quantum information technology [23,24,25,26,27,28,29]. A precise control and tuning of the QDs for various applications is however only possible through a detailed understanding of the growth mechanism at the atomic level, which creates the need for atomic-scale structural and compositional characterization. In this review we present the results of detailed structural and composition analysis by cross-sectional scanning tunneling microscopy (X-STM) and atom probe tomography (APT) of self-assembled QDs grown by droplet epitaxy where we focus mainly on X-STM and APT characterization of strain-free GaAs/AlGaAs and strained InAs/InP QDs grown by droplet epitaxy.

## 2. Self-Assembled III-V Semiconductor Quantum Dots

In this section, the two growth mechanisms of self-assembled QDs are briefly explained along with different techniques for the characterization of QDs. Typical III-V semiconductor QDs are grown either by conventional molecular beam epitaxy (MBE) or metal-organic vapor phase epitaxy (MOVPE). In the early 1990s, mainly two growth mechanisms were proposed for the fabrication of self-assembled QDs: (i) Stranski-Krastanov growth mode and (ii) droplet epitaxy. Both growth modes are still being explored to further optimize the nanostructures for various novel applications.

**Figure 1:**
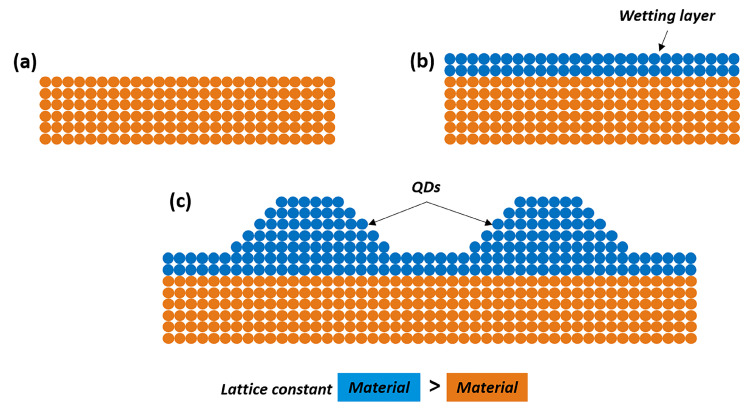
Schematic process showing the formation of SKQDs: (**a**) buffer layer; (**b**) formation of wetting layer; (**c**) QD formation. QD formation is decided by the lattice-mismatch between the two materials.

In the SK growth mode, strain induced formation of QDs occurs due to the lattice mismatch between the substrate and the epitaxially grown layer. Initially a two dimensional (2D) wetting layer of the same material as QD is formed, after reaching a critical thickness of typically 1–2 monolayers (MLs), the growth deviates from a layer-by-layer growth and three dimensional (3D) islands are formed to accommodate the local strain. A schematic process of SKQDs formation is shown in Figure 1. The most typical material system used for the formation of QDs by the SK-growth process consists of a few monolayers of In(Ga)As deposited on a GaAs substrate. In(Ga)As has a higher lattice constant than GaAs giving rise to a lattice mismatch of ∼7%, when grown epitaxially on a GaAs substrate. Initially, InAs forms a wetting layer on GaAs as growth evolves layer by layer. After reaching a critical thickness of ∼1.7 MLs for the case of pure InAs deposited on GaAs, the stored strain energy due to the lattice mismatch changes the growth morphology from layers to islands. The critical thickness or critical strain is a function of composition of the epitaxial layer. The strain relaxation can also lead to the formation of crystal defects, which strongly affect the optoelectronic properties of the QDs [30,31,32,33]. Developments in the growth techniques such as MBE or MOVPE in combination with decades of growth optimization made it possible to realize (nearly) defect free QDs. Despite the huge success of this growth technique, which has been used to create QDs for various optoelectronic applications such as lasers and quantum information technologies [9,10,28], the available degrees of freedom to control the QD formation are limited. The SK-growth mode is only available for lattice-mismatched material systems and the presence of 2D wetting layer coupled to the QDs is almost inevitable. The size and shape of the QDs mainly depends on initial layer thickness, growth temperature and the lattice mismatch. Major constraints of SK-growth mode includes: the presence of 2D wetting layer, residual strain fields, strain driven intermixing of QDs with capping layer leading to variety of composition profiles, energetically favored evolution of QDs with specific facets and dimensions, high aspect ratio (base to height),etc. The constraints of SK-growth can be largely eliminated by the fabrication of self-assembled QDs through droplet epitaxy.

**Figure 2:**
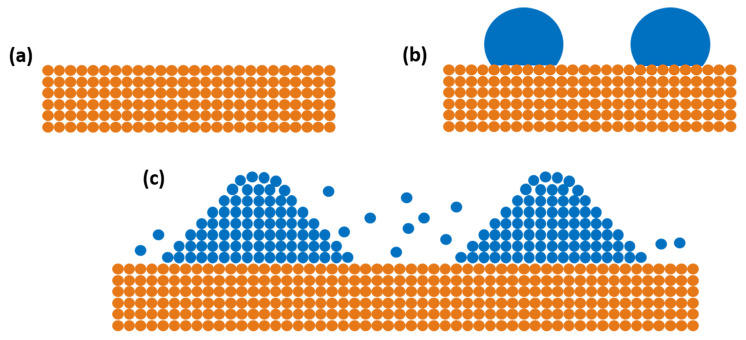
Schematic process showing the formation of DEQDs: (**a**) buffer layer; (**b**) deposition of group III droplets on the surface; (**c**) crystallization of formed droplets in group V rich environment.

Droplet epitaxy (DE) involves the formation and crystallization of metallic droplets to form QDs. DE was first proposed by Koguchi et al. [34], where they presented a new growth mechanism by splitting the group III and V supply in the MBE growth chamber. The fundamental step in DE is the formation of group III droplets (Ga/In), which allows the independent control over size and density of the QDs. The droplet formation can be controlled by optimizing the group III molecular beam flux and the substrate temperature to obtain desired size and density of the QDs. The surface reconstruction of the growth surface prior to the droplet deposition strongly influence the formation of nanostructures [35,36]. Later, the formed droplets are crystallized in group V (As) rich environment forming the QDs, as shown schematically in Figure 2. The dissolution and adsorption of group V element by the droplet and the surrounding surface govern the process of DEQDs formation. The crystallization kinetics depending on group V flux and crystallization temperature play a crucial role in determining the final shape (dots, disks, rings) and composition of the QDs [35]. Unlike in SKQDs, DEQDs can maintain their shape even after capping due to the reduced intermixing, which is largely driven by the lattice mismatch, between QDs and the capping layer. The DE growth mode has many advantages compared to SK growth mode: ability to grow QDs without a wetting layer [37], independent control over QDs size and density during droplet deposition, both lattice matched [38] and lattice mismatched [39] materials can be grown by DE, precise control over shape engineering allows the formation of complex nanostructures. It is also possible to grow QDs via heterogeneous droplet epitaxy where two group III atoms are supplied simultaneously to form droplets and later crystallized to form QDs [40,41]. A detailed review on droplet epitaxy growth and optimization for various nanostructures can be found elsewhere [42,43,44,45].

## 3. Characterization Techniques

Because the properties of both SKQDs and DEQDs are strongly influenced by their size, shape, and composition it is important to understand the growth mechanism at the atomic level and to analyze the effect of growth conditions on QD growth. Detailed atomic-scale characterization of QDs is thus necessary to improve their optoelectronic properties. Different characterization techniques that can be employed to resolve the size, shape and composition of the QDs are described in this section. Each characterization technique has its own advantages and limitations in providing structural and composition information of the QDs.

### 3.1. Atomic Force Microscopy

Atomic force microscope (AFM) invented by G. Binnig et al. [46] in 1986 is one of the scanning probe techniques with image resolution in the nanometer scale. AFM consists of a cantilever tip attached to a piezo-actuator to scan across the surface. The atomic forces between the tip and the surface causes a measurable deflection of the cantilever which is used to reproduce the surface topography. Generally an optical lever method is used to detect the cantilever deflections, where a laser beam is focused on the back side of the cantilever and the reflected beam is collected by a photodiode. The laser beam deflection system is connected to a feedback loop to control the force and the position of the tip. AFM can be operated in three different modes: (i) contact mode, where the tip is continuously in contact with surface; (ii) non-contact mode, where tip oscillates above the surface and the forces were measured; (iii) tapping mode, where the tip contacts the surface intermittently oscillating close to the resonance frequency. At short distances away from surface the van der Waals forces are present which attract the tip toward the surface. When the distance of the tip from the surface is further decreased, the repulsive forces due to the interaction between electronic clouds of tip and the surface become dominant. A detailed review on AFM was reported in Ref. [47,48].

**Figure 3:**
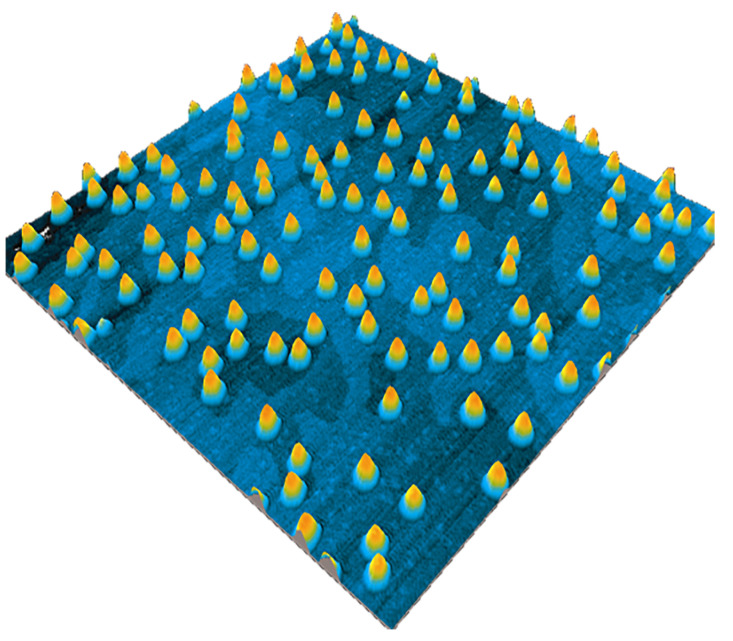
1 × 1 μm${}^{2}$ atomic force microscopy (AFM) image of InAs quantum dots grown on a GaAs surface by Stranski-Krastanov growth mode. Atomic steps on the GaAs surface can be seen in between the QDs. Adopted from Ref. [49].

AFM is extensively used to characterize QDs on the growth surface at various stages of growth. The uncapped QDs are characterized by AFM to provide details on size, shape and density of the QDs, which is essential information for growth optimization. An AFM image of InAs SKQDs grown on GaAs is shown in Figure 3, where the QDs are presented as 3D islands and the atomic steps on GaAs surface can also be identified in between the QDs.

Although AFM is a quite successful as a quick and first hand characterization technique to provide details about the QDs geometry and density, it has certain limitations. AFM can only provide structural analysis of uncapped QDs such as size and shape. However for practical applications, the QDs must be embedded in a matrix. It has been shown that significant changes in the morphology and composition can occur during the overgrowth [50]. More importantly AFM cannot provide any estimation of composition of the QDs, for which other techniques are necessary.

### 3.2. Transmission Electron Microscopy

**Figure 4:**
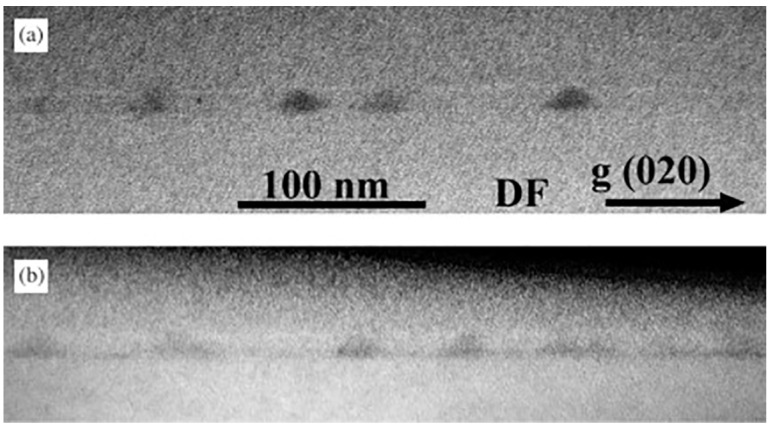
Dark field cross-section TEM images of GaAs/AlGaAs DEQDs, taken along the [0 2 0] axis: (**a**) sample A: QDs without WL; and (**b**) sample B: QD with 3 MLs WL; Reprinted from Ref. [37] © 2003, with permission from Elsevier.

Transmission Electron Microscopy (TEM) is a high resolution imaging technique in which an electron beam is transmitted through a thin sample. The interaction of transmitted electron beam with the sample produces the image. The electron source and the detector are located on opposite sides with respect to the sample. The e-beam transmits through the sample and then is captured and processed by the detector(s). The interaction volume of the e-beam is very small and equivalent to the de Broglie wavelength of the electrons. In principle, TEM can identify the position of lattice planes in a crystalline solid and can produce the lattice diffraction pattern as well. Additional features are available in TEM: an electron beam is swept in a raster over the sample producing a scanning transmission electron microscopy (STEM) image; Energy dispersive X-ray spectroscopy (EDX) can be used to estimate the composition of the sample [51]. The cross-sectional variant of the TEM (X-TEM) is capable of imaging embedded QDs to provide both structural and compositional insight. Figure 4 shows two dark field (DF) X-TEM images of GaAs/AlGaAs QDs grown by droplet epitaxy with and without wetting layer formation. The QDs can be identified by the dark pyramid like features in the bright matrix. In a special study electron microscopy has been used to create a 3D tomographic image of a single QD [52].

However, sample preparation and realization of proper thinning for TEM analysis is highly complex and time consuming. During the preparation of a thin lamellae of the sample by using micro and nano-machining techniques or using focused ion beam (FIB), there is a high probability to modify or damage the structure of the sample. The local information is limited and the obtained results are averaged over the whole lamellae where also local strain fields can affect the image contrast. Additional techniques are needed to derive the local fluctuations in the morphology and composition at the atomic scale.

### 3.3. Atom Probe Tomography

Atom Probe Tomography (APT) is a combination of field ion microscope with a spatially resolved time of flight mass spectrometer [53]. In APT, high-voltage pulses are applied to a sharp needle shaped sample to induce the emission of single ions from the apex which are accelerated towards a detector. The data from each voltage pulse is gathered to form a 3D topographic image of the sample. This technique is best suited for high conductivity material such as metals where a subatomic resolution can be obtained. Laser based APT extended the range of materials that can be analyzed by APT [54]. Additional thermal excitation and photoconductivity induced by the pulsed laser made it possible to perform APT on semiconductor nanostructures containing for instance QDs [55,56]. APT is capable of providing complete three dimensional reconstruction of the topography along with mass spectral analysis to identify different chemical species. Alongside its strengths to provide atomic data, APT has certain limitations. The measurable volume in APT is typically limited to few hundreds of nanometers (nm) vertically and a few tens of nm in the lateral direction. The detection efficiency is in the order of 60% even under ideal conditions [57]. The sample preparation is a complex process involving FIB to make the tips for APT analysis. Image distortions during reconstruction due to the many constraints and assumptions, ion identification ambiguity for some complex systems etc. A detailed history and advancements of APT can be found elsewhere [58,59] and an example of QD analysis by APT is reported in [55].

### 3.4. Cross-Sectional Scanning Tunneling Microscopy

Scanning tunneling microscope (STM) invented by Binnig and Rohrer in 1981 [60,61,62] is an imaging technique that can achieve atomic resolution. It works on the principle of quantum mechanical tunneling through a vacuum barrier. STM consists of a sharp metallic tip scanning over the surface with the help of piezoelectric stacks. A bias voltage is applied between the sample and the tip and when the tip is close enough to the surface, a measurable tunneling current is detected. At negative sample bias, the electrons tunnel from filled states of the sample to the tip (filled-state imaging) and at positive bias voltages the electrons tunnel from tip to the empty states of the sample (empty-state imaging). The tunneling current is extremely sensitive and exponentially decays with the tip-sample distances. STM can be operated in two different modes: (i) Constant current mode: where the tunneling current is kept constant and the differences in height are measured; (ii) Constant height mode: the height of the tip is kept constant and measuring the tunneling current. A feedback loop is employed to maintain either constant height or constant current and to adjust the tip-sample distance to maintain either constant current or constant height.

In cross-sectional STM (X-STM), the images are obtained at the cleaved facet of a crystalline material. This was used for the first time successfully in the study of the GaAs {110} surface [63,64]. X-STM has shown to be an excellent tool to probe semiconductor nanostructures at the atomic scale, especially in the case of III-V semiconductor materials. III-V semiconductors have a charge neutral {110} natural cleaving plane, which makes it possible to obtain atomically flat surfaces for X-STM analysis. The X-STM measurements are performed on a clean {110} surface freshly obtained by cleaving the sample in ultra-high vacuum (UHV). In filled-state imaging at high negative bias voltages group V sublattices are imaged, while in empty-state imaging at positive bias voltages group III sublattices are imaged. Due to the atomic arrangement of the {110} surfaces of Zinc-blende crystals, only every second monolayer along the growth direction is visible in the X-STM images [65]. STM-tips are made of polycrystalline tungsten wires obtained by electrochemical etching followed by baking and Ar sputtering inside the STM preparation chamber in UHV. X-STM is capable of resolving semiconductor nanostructures with atomic resolution. The precise structural analysis is essential for the better understanding of growth mechanisms and to optimize QDs for various optoelectronic applications [9,10,28]. X-STM can directly visualize the atomic structure of the surface and so precisely determining the size and shape of the embedded QDs. The structural and compositional changes after overgrowth such as intermixing, segregation and morphological changes in QDs can also be studied by X-STM [50,66,67,68,69,70,71,72,73,74,75,76,77,78]. The cleaving of the sample produces an outward relaxation due to the compressively strained QDs and this can be experimentally measured by X-STM. The atomic resolution structural analysis from X-STM can be combined with finite element simulations to estimate the composition of the nanostructures [50]. The first X-STM analysis of QDs grown by a droplet epitaxy related technique was reported in Ref. [41]. Not only structural information can be obtained by X-STM but also electronic effects, such as wavefunctions of confined states, can be imaged [79,80,81,82]. Although X-STM is capable to deliver excellent atomic resolution due to the fact that the tunneling process is only sensitive to a single (surface) layer intersecting the QD, it is unfortunate that the cleavage process randomly intersects a QD and thus no 3D information of the same dot can be obtained by X-STM. Unfortunately the application of X-STM has been less successful for non-III-V materials.

## 4. Strain-Free GaAs/AlGaAs DEQDs

The optoelectronic properties of QDs are strongly affected not only by size, shape and composition but also by strain fields in the vicinity of the QDs which strongly affect the electronic eigenstates of the QDs. Since, the SKQDs are formed due to the lattice mismatch, the strain is inevitable in the self-assembled SKQDs. GaAs and AlGaAs have a similar lattice constant making it impossible to grow GaAs QDs in SK mode. Interestingly, droplet epitaxy is capable of fabricating strain-free GaAs QDs on AlGaAs [83,84,85,86]. Initially an AlGaAs buffer layer is grown on top a GaAs substrate at a temperature of 580–600 °C. Later, the As is evacuated from the growth chamber while lowering the substrate temperature to 200–400 °C for Ga droplets deposition on top of the AlGaAs. Both size and density of QDs can be optimized by controlling the Ga flux and the substrate temperature. The Ga droplets are crystallized in As rich environment to form the GaAs QDs, followed by AlGaAs overgrowth. The final morphology of the QDs is strongly influenced by the crystallization temperature and the amount of As flux. Partial capping followed by annealing and flushing techniques can be employed to further optimize the nanostructures. In this section we present a detailed review on structural and compositional analysis of strain-free GaAs/AlGaAs QDs by X-STM and APT [73,77].

### 4.1. Size and Shape of the QDs

**Figure 5:**
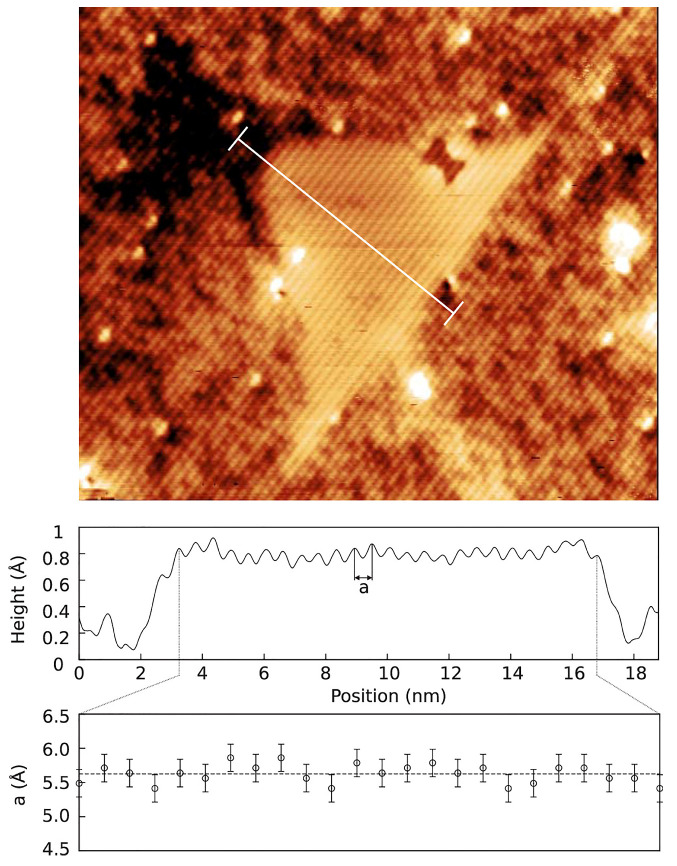
40 × 34 nm${}^{2}$ topographic image of a typical GaAs/AlGaAs QD (**top**) taken at bias voltage $V_{b}$ = –3 V and $I_{t}$ = 40 pA; An average cross-sectional profile (**top graph**) and separation between bilayers (**bottom graph**) along the line in the top figure. Reprinted from Ref. [73], © 2010 with the permission of AIP Publishing.

Figure 5 shows a filled-state topographic image of a typical GaAs DEQD in AlGaAs with well-defined interfaces and dimensions of height ∼13 nm and base length ∼28 nm. The AFM experiments on uncapped QDs grown under similar growth conditions obtained a similar QD dimensions [37,87].

The brightness in the image is due to the electronic contrast, where Al-rich areas give rise to a dark contrast and Ga-rich areas to a bright contrast. The clean bright region represents the pure GaAs and the mixed contrast is due to AlGaAs matrix. Another striking feature in the image is the presence of AlAs-rich region just above the QD (dark). This is due to the difference in the surface mobility of Ga and Al atoms, as Ga atoms are more mobile, they migrate more easily along the edge of the QD during the capping and the less mobile Al atoms remain closer to the top. The convex curvature of the growth front at the position of the QDs is the driving force behind the migration of the incoming adatoms away from the top of the QD [88]. The white line drawn through the center of the QD corresponds with the topographic profile plotted in the first panel below the X-STM image. The GaAs/AlGaAs QDs are expected to be strain free which is nicely confirmed by the absence of outward relaxation of the cleaved surface unlike in lattice-mismatched QDs [50]. The strain-free nature of the QD is further supported by lattice constant measurement in the second panel of Figure 5. The measured lattice constant is close to 0.565 nm (dashed line) indicating that the QD is indeed strain-free.

The effect of Al intermixing in the formation of GaAs QDs is one of the concerns frequently questioned in the literature [37,89].

**Figure 6:**
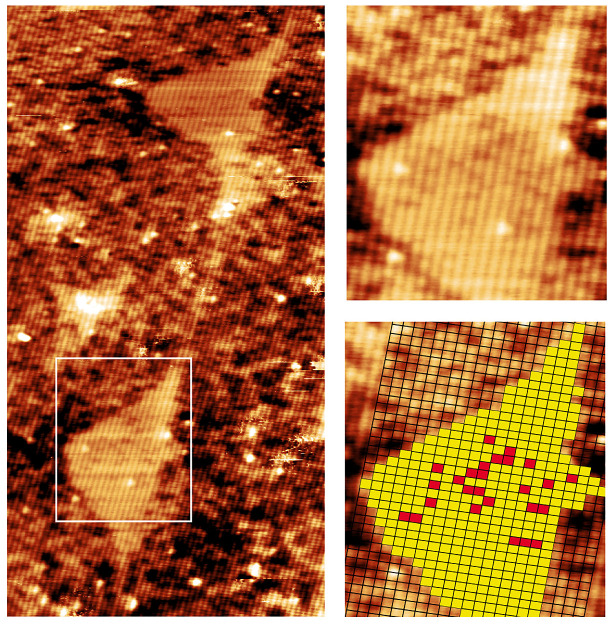
30 × 60 nm${}^{2}$ topographic image (**left**) of two QDs taken at $V_{b}$ = –3 V and $I_{t}$ = 40 pA. An atomic grid is overlain on top of a close up of the QD dot (**right**). Al and Ga atoms in the QD are indicated by red and yellow square respectively. Reprinted from Ref. [73], © 2010 with the permission of AIP Publishing.

Figure 6 shows a topographic image of two QDs with few dark spots in the QDs indicating Al intermixing. To perform a quantitative analysis, a grid with atomic dimensions was overlaid on top of one of the QDs (right), showing Al atoms in red and Ga atoms in yellow. A maximum concentration of 6% of Al was estimated in this particular QD where the level of Al intermixing changes from dot to dot, and plays a minor role in the formation of GaAs DEQDs. The QDs are found to be Gaussian in shape quite different from typical SKQDs which are truncated pyramid shaped [90]. The side facets of the QDs are not straight but found to be in the range of 33–55°, in which the upper bound corresponds to {111} facet (54.7°). The uncapped GaAs QDs were found to have {111} facets [83,91] so, it is possible that the shape of the QDs is slightly modified during the overgrowth. A wetting layer with a thickness less than a bilayer (BL) can be identified in Figure 6 in between the QDs. The formation of wetting layer strongly depends on the surface reconstruction of the growth surface prior to the droplet deposition. QDs grown on an arsenic stabilized c(4 × 4) reconstructed surface yields a wetting layer with a thickness of ∼1.75 MLs which corresponds well with the presented X-STM results [83].

### 4.2. GaAs-Rich Intrusions and Al Intermixing

**Figure 7:**
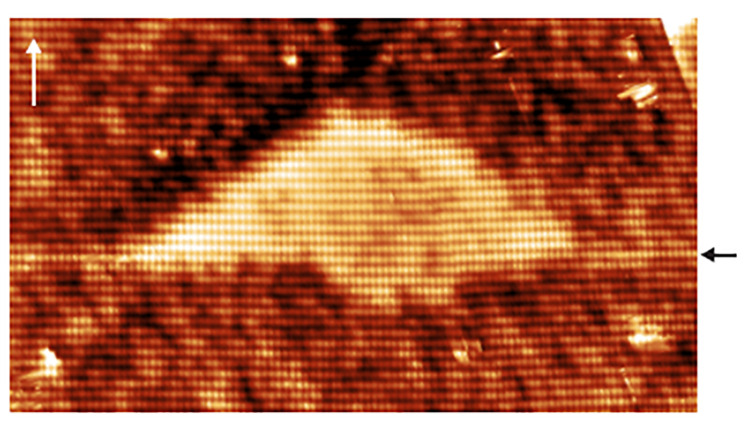
38 × 23 nm${}^{2}$ topographic image of a QD with a GaAs rich etch pit in the AlGaAs buffer layer, taken at $V_{b}$ = −3 V and $I_{t}$ = 40 pA. The growth direction and the position of the wetting layer are marked by white and black arrows respectively. Adopted from Ref. [93].

In Figure 6, one can observe a GaAs rich intrusion in the underlying AlGaAs matrix. These intrusions or etch pits are nearly always present in the QDs and show considerable degree of Al intermixing. Figure 7 shows a filled-state topographic image of another QD with a GaAs etch pit in the AlGaAs buffer layer. The formation of GaAs intrusions is a result of local etching process at the solid-liquid interface [92]. Local etching involves the diffusion of As atoms from the buffer layer into the Ga droplet, liquefying the buffer layer underneath the droplet.

This liquefaction of the buffer layer, causes the dissolution of Al atoms into the droplet. This is supported by the spatial correlation between the location of the etch pit and the region of Al intermixing. The dissolved As atoms are assumed to diffuse and crystallize at the QD edges, while the Al atoms do not have sufficient time to diffuse to the edges before the complete crystallization of the droplet. This observation was further supported by the photoluminescence and AFM studies on quantum rings (QRs) formed by local etching method [94].

**Figure 8:**
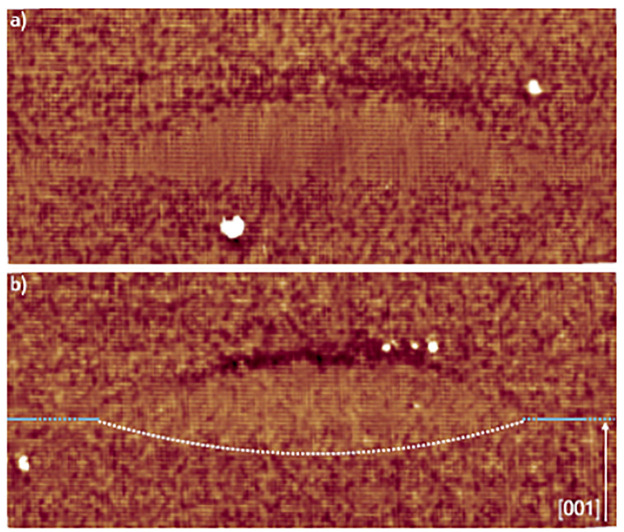
32 × 76 nm${}^{2}$ filled-state topography images of DE-QDs grown under the exact same conditions. Examples of QDs with (**a**) low and (**b**) high level of Al intermixing. The dotted line indicates roughly the bottom interface of the QD. Adopted from Ref. [95].

Filled-state images of two DEQDs grown under exact same conditions are given in Figure 8, where the effect of droplet etching is clearly visible. The QD with the bottom interface etched into the AlGaAs matrix shows high level of Al intermixing than the other QD. This observation supports the correlation between droplet etching and Al intermixing and the random occurrence of the intermixing process in the QDs.

**Figure 9:**
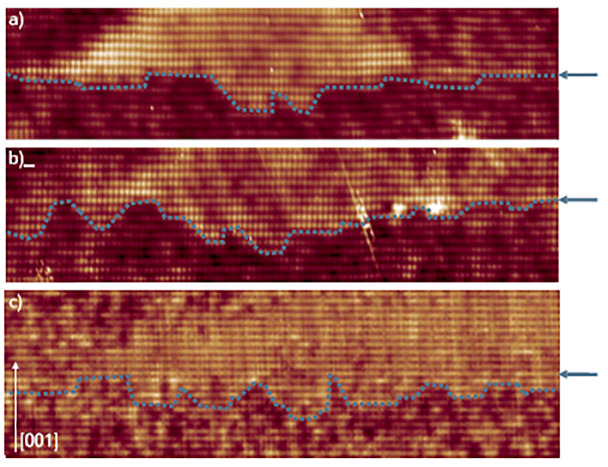
(**a**,**b**) 24 × 9 nm${}^{2}$, and (**c**) 35 × 13 nm${}^{2}$ filled-state topography images of the bottom interfaces (indicated by dotted lines) of DE-QDs. GaAs inclusions are present below the QDs. Locally above those intrusions more Al atoms are incorporated inside the QD. The arrow indicates the growth direction [001]. Adopted from Ref. [95].

Figure 9 presents the filled-state images of the bottom interfaces of three GaAs/AlGaAs QDs. It is often seen that the QD bottom interfaces is not well-defined, the interface roughness indicated by the dotted lines shows this point. The depth of these intrusions ranges from one bilayer to a few nanometers. The fluctuations in the QDs height due to the etch pits can strongly influence the optoelectronic properties, thus the growth conditions must be modified to reduce the formation of intrusions and Al intermixing.

### 4.3. Size Control and Composition of GaAs/AlGaAs DEQDs

**Figure 10:**
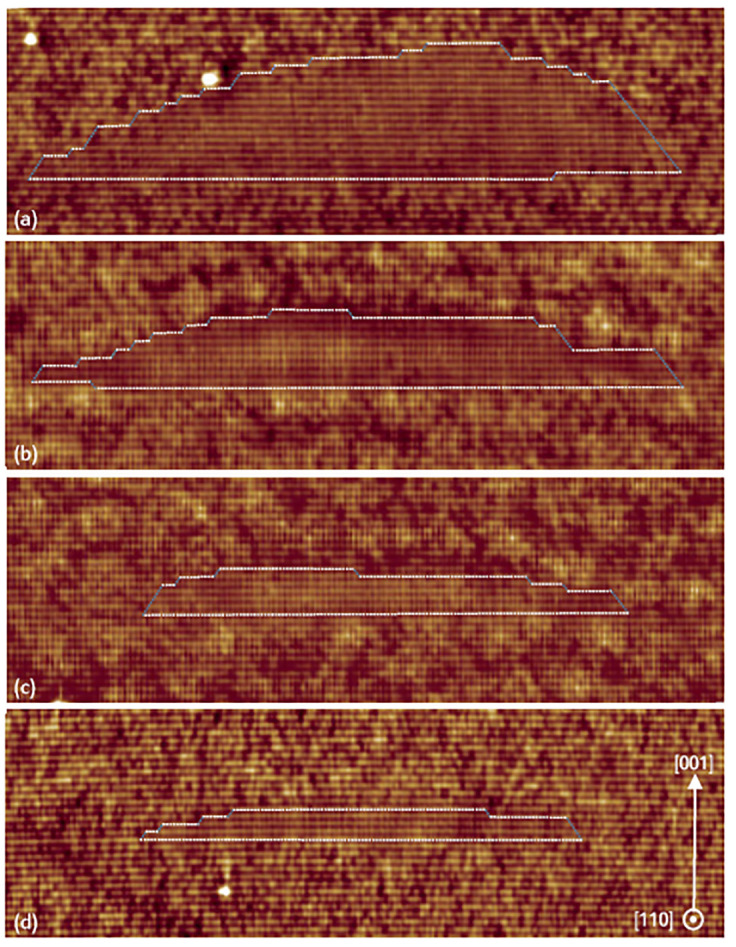
53 × 17 nm${}^{2}$ filled-state topography images of QDs for (**a**) 5 MLs, (**b**) 3 MLs, (**c**) 2 MLs and (**d**) 1.5 ML of Ga. The images correspond to 2D cuts close to the center of the QDs. The (110) facets are outlined in white and (111B) facets are outlined in blue. Reprinted figure with permission from Ref. [96] © 2015 by the American Physical Society.

As mentioned before, the size and density of QDs can be tuned by optimizing the substrate temperature and the amount of Ga flux supplied for droplet formation. The effect of the Ga flux on the size of the QDs is described in Figure 10. Four QD layers were grown changing the volume of deposited Ga from 1.5 ML to 5 MLs.

The QD height of 12 MLs (3.4 nm), 16 MLs (4.5 nm), 26 MLs (7.3 nm) and 44 MLs (12.4 nm) can be respectively attributed to 1.5 ML, 2 MLs, 3 MLs and 5 MLs of Ga deposited. The base length of the QDs was found to increase with the amount of Ga from 35 nm for 1.5 MLs of Ga to 50 nm for 5 MLs of Ga. However, the variation in height of the QDs is stronger than the variation in base length. Facet determination is quite challenging in these images due to the weak interface contrast. As reported in the literature [97], the GaAs DEQD facets can be described by both the (001) facets (white lines) and (111) facets (blue lines). As one can see, the (001) facets are wider for low Ga flux (1.5 and 2 MLs) than the high Ga flux QDs.

In Section 4.1, it was mentioned that the difference in the mobility of Al and Ga atoms at the growth front is responsible for the formation of AlAs-rich (dark area in Figure 5) region just above the QDs. The formation of such AlAs-rich region is observed only for the QDs grown from high Ga volume droplets. Up to 70% of these Ga depleted regions are formed in larger QDs (3 MLs and 5 MLs of Ga flux) and it was completely suppressed in smaller QDs grown from ≤2 MLs.

**Figure 11:**
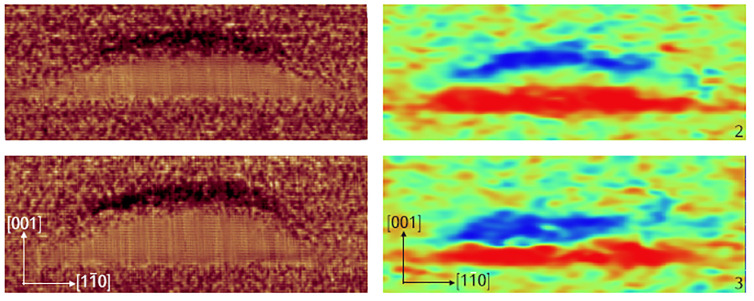
75 × 28 nm${}^{2}$ filled-state topography images of 2 different DEQDs grown from 5 ML of Ga (**left panel**). 60 × 30 nm${}^{2}$ 2D Al concentration maps (**right panel**) taken on one QD grown from 3 ML of Ga at different positions. The color scale is linear from 0% (red) to 35% (blue) of Al. An Al-rich region is present above the QD. Adopted from Ref. [95].

In the left panel of Figure 11, X-STM filled-state topographic images of two QDs grown from 5 MLs Ga flux are given showing the presence of AlAs-rich (dark area) regions just above the QDs. Similar effects are visible in the APT 2D concentration profiles (images on the right) of a single QD grown from 3 MLs of Ga taken at different positions. The Ga rich QD region is shown in red while the blue cloud above the QD indicates the Al rich area.

**Figure 12:**
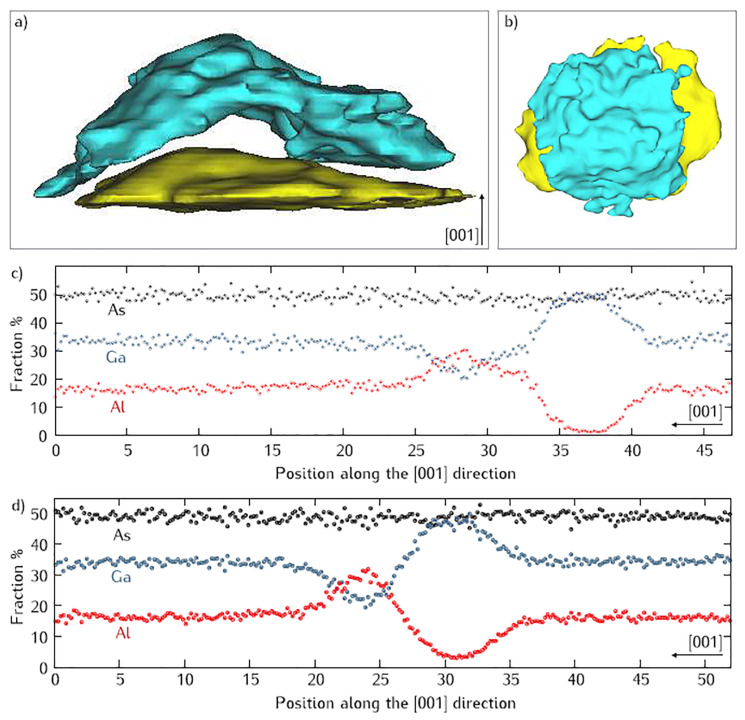
88% Ga iso-surface (yellow) and 50% Al iso-surface (blue) in a 3D APT data set, outlining a QD grown from 3 MLs of Ga, and its Al-rich cap: (**a**) 40 × 20 nm${}^{2}$ side view; (**b**) 50 × 50 nm${}^{2}$ top view; Ga and Al concentration profiles along the [001] direction for a QD grown from (**c**) 3 ML of Ga and (**d**) 5 ML of Ga. The Al-rich cap is separated from the pure GaAs by an intermixed region. Adopted from Ref. [95].

A 3D representation of APT data is displayed in Figure 12, only the upper part of the QD is covered with this AlAs-rich region. It is evident from 3D APT data that the anisotropy of this AlAs-rich region follows the anisotropy of the QD. The extent of Ga depletion can be estimated quantitatively by measuring 1D composition profiles along the growth direction [001]. Composition profiles measured on DEQDs grown from 3 MLs Ga and 5 MLs Ga are shown in Figure 12. Both profiles looks very similar and show that the QDs have an almost pure GaAs composition with an Al concentration of less than 5%. The broadening of top and bottom interfaces is confirmed by the absence of step like transition, which ranges from a few MLs to several nm depending on the QDs. AlAs-rich region can be seen in both profiles and the amount of Al increases to 60% in that region while Ga drops to 30%. The original AlGaAs matrix is made of 67% Ga and 33% Al, therefore the Ga/Al ratio is almost reversed in the AlAs-rich region, this inversion is clearly visible in the composition profiles. Comparing both X-STM and APT results, it appears that the AlAs-rich region is not directly in contact with the QD. It seems that there is a thin layer of AlGaAs in between the QD and the AlAs-rich region.

The size control of DEQDs can also be obtained through engineering the capping layer. This method of optimizing QDs structure was first demonstrated in strained SKQDs, by changing capping layer composition [31,98,99,100], capping rate [101], strain field [20], adding growth interruptions and annealing steps [102,103]. Such a capping process (flushing technique) can be applied to GaAs/AlGaAs DEQDs to obtain a precise control over QDs height. In flushing process, the QDs are grown by following the standard droplet epitaxy procedure. After crystallization, the DEQDs are partially capped with AlGaAs layer at intermediate temperature in which the capping layer thickness is chosen to be lower than the expected height of the QDs. This leads to partial capping of QDs and exposes the top part of the QDs. During this growth interruption, an As rich environment is maintained while increasing the substrate temperature. This step favors the dissolution of exposed top facet of the GaAs QD which results in a truncated shape. Later the QDs are capped with AlGaAs layer at intermediate temperatures followed by annealing at nominal growth conditions.

A sample with four layers of QDs was studied by X-STM to verify the efficiency of flushing process on size optimization of the DEQDs.Among the four layers, the first QD layer was grown with standard DE conditions without flushing as a reference for comparison. Second and third layers were grown with a partial capping of 2 nm and 4 nm respectively. The last one is grown on top of a 2 nm GaAs quantum well (QW) with 4 nm AlGaAs capping layer. All other growth conditions are kept constant for all four layers, Ga volume of 5 MLs, crystallization temperature of 170 °C at an As flux of 1.2 × 10${}^{-4}$ Torr. In between the two AlGaAs capping steps, the layer was annealed at 640 °C to dissolve the exposed top facet from partial capping.

**Figure 13:**
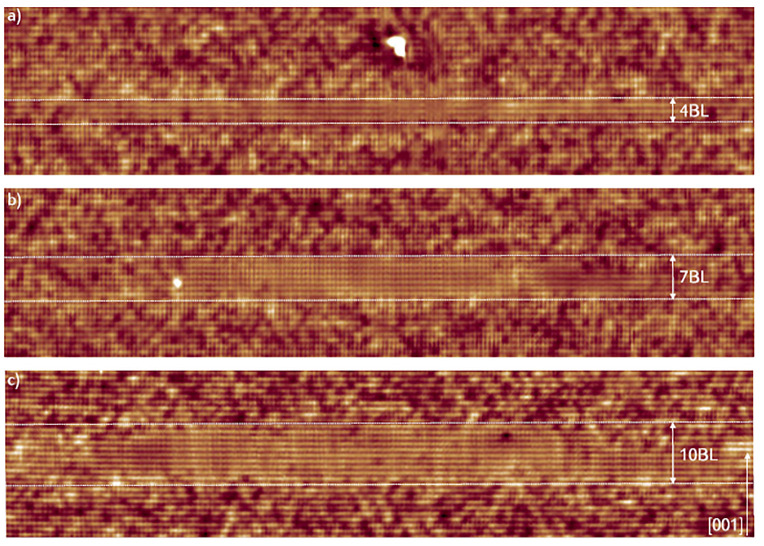
68 × 15 nm${}^{2}$ filled-state topography images of DE-QDs grown with the flushing procedure. (**a**) 2 nm AlGaAs partial capping. (**b**) 4 nm AlGaAs partial capping. (**c**) 4 nm AlGaAs partial capping + 2 nm GaAs QW. Adopted from Ref. [95].

Filled-state topographic images of the DEQDs grown with the flushing step are given in Figure 13. The position of top and bottom interfaces are indicated by white dotted lines along with the height of the QDs in BLs. The QDs grown with flushing process appear to be more homogeneous and the degree of intermixing is lower than the reference QDs (X-STM images in Figure 11). The Ga-rich intrusions cannot be distinguished at the bottom interface, however, Al atoms can be identified in the QDs close to the bottom interface. The top interface of all the QDs is found to be abrupt where the transition from GaAs/AlGaAs occurs within a BL. The truncation of QDs top facet by flushing is the main reason for the sharpness of top interface. Growth of GaAs DEQDs on a GaAs QW mainly increases the base diameter of the QDs, this is due to the spontaneous inter-diffusion process at the GaAs/AlGaAs interface. The quality of top interface demonstrates the efficiency of flushing process. One can easily notice the absence of AlAs-rich regions on top of all the flushed QDs. Application of flushing technique makes it possible to obtain DEQDs with desired height, low level of Al intermixing, and reduced Ga-rich intrusions.

### 4.4. GaAs/AlGaAs Quantum Dots and Quantum Wires on GaAs(311)A Substrate

The modification of QDs size and density can also be obtained by switching to higher miller index substrates such as GaAs (311)A. The highest obtainable QDs density through droplet epitaxy on a GaAs (001) substrate is close to ≈2 × 10${}^{10}$ cm${}^{-2}$, which is comparable to the density of SKQDs. However, the lower limit of density puts bounds on applicability of DEQDs in some optoelectronic applications such as lasers, where high density is desirable. Growth on (311)A substrates is a promising technique to obtain densities beyond 10${}^{11}$ cm${}^{-2}$ due to the reduced Ga migration length [85,104]. On top of that, fabrication of quantum wires (QWRs) is possible due to the anisotropic nature of the (311)A surfaces [105]. These QWRs are highly asymmetric nanostructures with optical properties that are beneficial in lasers [106] that use cleaved {110} surfaces as Fabry-Pérot mirrors. A sample with one GaAs/AlGaAs QWR layer and one GaAs/AlGaAs QD layer grown on top of a n-doped GaAs (311)A substrate is investigated by X-STM. 5 MLs of Ga is deposited to form QDs, after which the droplets are annealed at 550 °C to form QWRs. In the same way, a second layer is grown by annealing at 400 °C to form QDs instead of QWRs. Detailed growth conditions and AFM analysis of uncapped QDs and QWRs are reported in Ref. [75].

**Figure 14:**
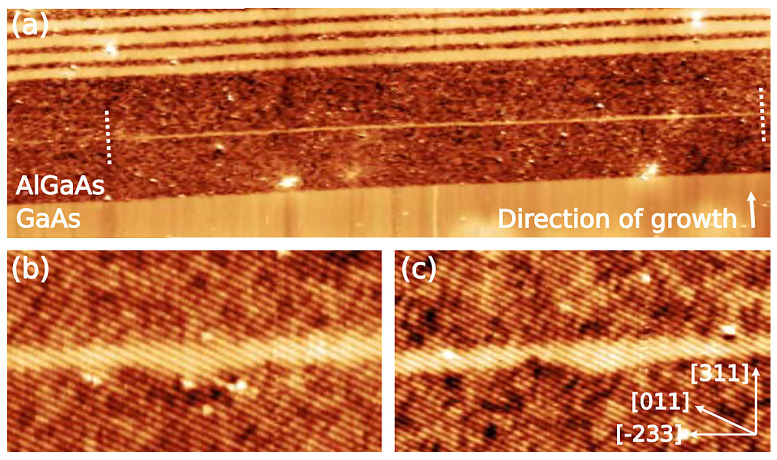
(**a**) 250 × 75 nm${}^{2}$ topographic X-STM image of one complete QWR (marked by the two dashed lines). (**b**,**c**) 30 × 16 nm${}^{2}$ close ups of the interface fluctuation. Reprinted from Ref. [75], © 2011 with the permission of AIP Publishing.

Figure 14a shows a filled-state topographic X-STM image of a full QWR (between the two white dotted lines) in the middle of AlGaAs matrix. The length of QWR is found to be 220 nm, the height of the QWR fluctuates between 1.3–1.9 nm. Typically, the QWRs are found to be longer than 250 nm, in agreement with the AFM analysis [75]. The distance between the QWRs in [$\bar{2}$33] direction is found to be in the order of tens of nanometers. Figure 14b,c shows the interface roughness of the GaAs QWR with AlGaAs matrix. The top interface looks very abrupt and this suggests that these intrusions at the bottom interface are formed prior to or during the droplet crystallization but not during the capping process. Based on the homogeneity of the color contrast within the QWR, it is clear that no Al intermixing is happening in the system.

**Figure 15:**
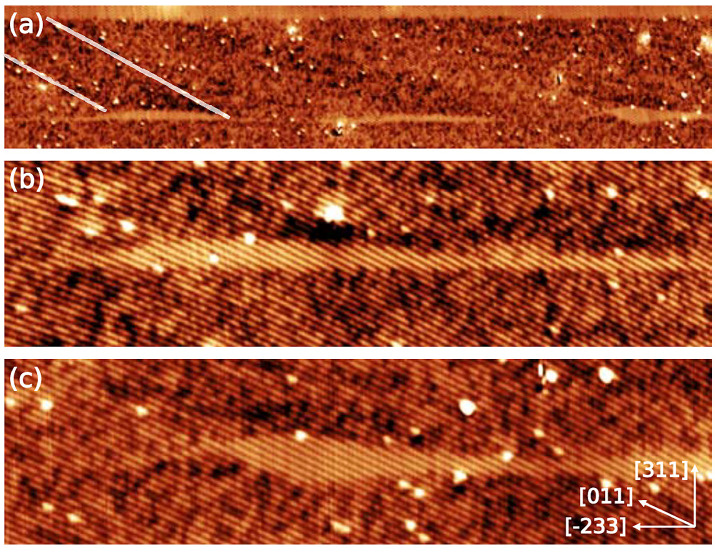
(**a**) 170 × 35 nm${}^{2}$ topographic X-STM image of the QD layer. An AlAs-rich region emanating from the top of one of the QDs is marked by two transparent lines; 65 × 16 nm${}^{2}$ topogrpahic image of a typical QD with a smooth (**b**) and rough (**c**) bottom AlGaAs/GaAs interface. Reprinted from Ref. [75], © 2011 with the permission of AIP Publishing.

A typical part of the QD layer is shown in Figure 15, where three individual QDs can be identified without any indication of the wetting layer. The average height of the QDs is estimated to be 2.3 ± 0.6 nm, which is in line with the AFM measurements indicating a limited structural change during capping. Figure 15b,c shows both the smooth and rough AlGaAs/GaAs interface at the bottom respectively. Due to the high dot density, most of the QDs are found to overlap with other QDs. The QDs consists of a (211) and (411) side facets. Due to the limited migration length of Al atoms, AlAs-rich regions are formed above the QDs. The continuous shift of the asymmetric QD shape at the growth front in [$\bar{2}$33] direction during the overgrowth results in a tilt of the AlAs rich region in [011] direction. Previously explained Ga-rich intrusions due to local etching [92,94] are also observed in both QWRs and QDs. All in all, growing nanostructures on (311)A oriented substrates is beneficial to obtain QWRs and QDs with minimal structural changes, high density, and reduced Al intermixing.

## 5. Strained InAs/InP DEQDs

Entangled photon emission from the QDs can be generated naturally with a small fine-structure-splitting (FSS) between the exciton eigenstates. Asymmetry in the QD wavefunction due to variations in size, shape, and composition of the QDs is a known source for FSS. The elongation of QD shape is a natural consequence of the SK growth technique. Various techniques can be used to obtain QDs with small FSS such as, growing QDs on (111) surfaces in which the underlying C${}_{3v}$ crystal symmetry assists in obtaining uniform QDs [107,108,109,110,111]. Fabrication of QDs in locally etched pits is another technique to reduce anisotropy in QDs size and shape [112]. Although entangled photon emission can be observed in QDs grown by these methods, the emission is below the conventional telecom region. The long distance quantum communication networks rely on the efficient single and entangled photon sources emitting at a relatively low-loss wavelength around 1.55 μm. InAs/GaAs QD systems emitting at ∼900 nm provided a physical system to demonstrate the basic building blocks of a quantum network, such as entangled photon pairs from electrically driven sources [11]. On the other hand, InP based QD systems can readily emit in the telecom wavelength range around 1.55 μm. The lower strain compared to InAs/GaAs QDs pushes the emission wavelengths to the conventional telecom band. The InAs/InP droplet epitaxy QDs grown by MOVPE emitting at ∼1.55 μm can be employed as both single photon and entangled photon emitters [8] with a mean FSS 4 times smaller than that of SKQDs [113].

### X-STM of Strained InAs/InP DEQDs

In this section we report on recent X-STM analysis performed on InAs/InP DEQDs grown by MOVPE using H${}_{2}$ as carrier gas. The growth started in a low-pressure reactor by the deposition of ∼2 MLs of In droplets on the InP surface via the pyrolysis of trimethylindium at 400 °C without supplying arsenic to the growth chamber. The chamber pressure is maintained at 150 Torr during this process. The deposition rate of In is equivalent to a growth rate of InAs of 0.04 nm·s${}^{-1}$. The crystallization of the QDs under arsine over pressure starts at 400 °C and carries on until the substrate reaches 500 °C. Later, the QDs are capped with 30 nm of InP followed by more InP at 640 °C. The precursors used in the growth are Trimethylindium-$In{\left(CH_{3}\right)}_{3}$, trimethylgallium-$Ga{\left(CH_{3}\right)}_{3}$, trimethylaluminium-$Al_{2}{\left(CH_{3}\right)}_{6}$, phosphine-$PH_{3}$, and arsine-$AsH_{3}$. Complete growth details and FSS experiments are reported in Ref. [113]. X-STM is performed at LN2 temperature (77 K) on a freshly obtained {110} surface obtained by cleaving the sample in UHV.

**Figure 16:**
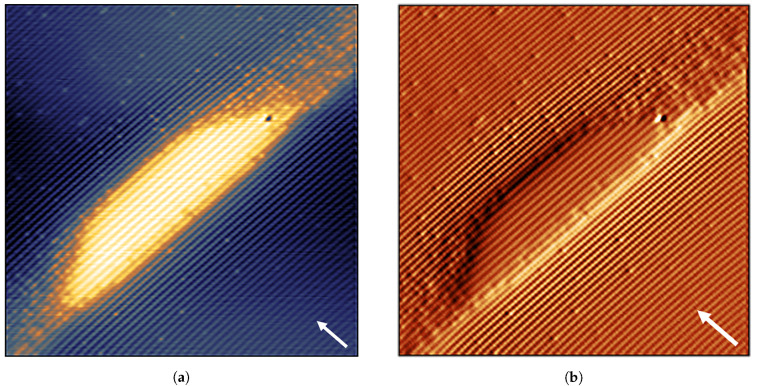
30 × 30 nm${}^{2}$: (**a**) filled-state topographic image (dark to bright contrast represents a height difference of 338 pm); (**b**) current image (bright to dark contrast represents current difference of 40 pA); taken at *V_b_* = −3 V, *I_t_* = 50 pA revealing the typical structure of the InAs/InP DEQDs. The arrow indicates the growth direction [001].

Figure 16 reveals the typical structure of the InAs/InP DEQDs grown by MOVPE. One can clearly observe the truncated pyramid shape of the QDs with well defined facets. The QD has a base length of 27 ± 0.5 nm with a height of 10.5 ± 0.5 nm (11 BLs). Even though the size distribution of DEQDs is superior to the SKQDs, there is still observable inhomogeneity in the size distribution of these InAs/InP DEQDs. The height and base length of uncapped InAs/InP QDs grown under similar conditions measured by AFM are reported in Ref. [113]. We observed a good agreement in dimensions of QDs measured by both AFM and X-STM, especially similar height of the QDs, indicating the absence of structural changes after the overgrowth. The color contrast in Figure 16a represents the relative height of the STM tip from the surface. The brightness in the X-STM topographic image is due to the relaxation of compressively strained InAs region after cleaving indicating the strained nature of QD system unlike the strain-free GaAs/AlGaAs DEQDs. The uniformity in the color contrast within the QD indicates a pure InAs QD without any intermixing. This is further supported by looking at the current image of the same QD (Figure 16b), where there are no observable fluctuations in the current response within the QD. This observation is true for all the QDs found during the measurement. Even though the shape of the QD is that of a truncated pyramid (typical shape of the SKQDs), the absence of intermixing demonstrates the benefit of droplet epitaxy to obtain pure QDs where intermixing is almost unavoidable in SKQDs [50]. STM height profile of the compressively strained InAs QD shown in Figure 16a is measured by taking a line profile through the center of the cleaved QD. The outward relaxation of almost 250 pm of the cleaved QD further supports the strained nature of the InAs/InP DEQDs. These InAs/InP dots look much more well-behaved concerning interface abruptness and dot shape than the InAs/GaAs droplet dots that were also studied by X-STM [114]. The larger mismatch between the dot and host material for the InAs/GaAs QDs and the fact that also these dots are also rather pure in InAs is most likely responsible for the growth instabilities affecting the final dot shape.

**Figure 17:**
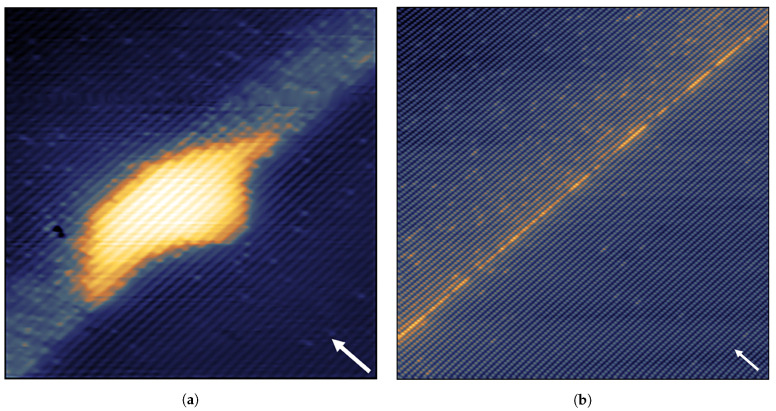
(**a**) 25 × 25 nm${}^{2}$ filled-state topographic image of the InAs/InP DEQD with a very deep etch pit (5 BLs); (**b**) 40 × 40 nm${}^{2}$ filled-state topographic image of the discontinuous wetting layer in InAs/InP DEQDs. The arrow indicates the growth direction [001].

Careful observation of the filled-state topographic image (Figure 16a), uncovers the InAs intrusions in the bottom InP layer, similar to the GaAs intrusions in AlGaAs layer reported in Section 4.2. The process of local etching is occurring at the interface between liquid In droplet and underlying InP layer. We observed the droplet etching in almost all the QDs observed during the X-STM experiment. The diameter of the etch pit ranges from 1 BL to few nanometers, depending on size of the QD and also the cleaving position. One of the biggest InAs etch pits found during the X-STM measurement is given in Figure 17a. The depth of etching extended up to 5 BLs into the underlying InP layer. The etch pit shows no preferential position and is randomly positioned at the bottom of the QD. There maybe a correlation between the size of the QD and size of the etch pit, as we observed bigger etch pits for smaller QDs. Comparing the etch pits of both GaAs/AlGaAs DEQDs and InAs/InP DEQDs, one can observe that the etch pits in InAs/InP QDs are very localized. These etch pits might influence the optoelectronic properties of the QDs as they might change the charge distribution of the carriers in the dot, especially the effect of these etch pits on single and entangled photon emission needs to be investigated both experimentally and theoretically. Another striking feature of DEQDs is the absence of wetting layer (WL) unlike in SKQDs where WL is inevitable. We observed a discontinuous layer of InAs(P) shown in Figure 17b. The estimated composition of the WL is $InAs_{x}P_{1-x}$, where *x* = ∼0.65 and thickness is less than a bilayer. The discontinuity of the WL is clearly visible in the X-STM image. The As-P exchange at the growth surface is most probably involved in the formation of discontinuities in the WL. It is important to note that there is no observable intermixing even in the etch pits, as there are no noticeable contrast fluctuations in the QD or in the etch pit. However, slight intermixing can be observed close to the QD edges but, the level of intermixing is very low compared to SKQDs. Thus, QDs grown by DE either strained or strain-free, are superior to those grown by SK-mode in terms of structural and optical quality. DE allows precise control over size, shape, composition, and density of the QDs.

## 6. Conclusions

In conclusion, we presented a detailed review on atomic-scale characterization of droplet epitaxy quantum dots by cross-sectional scanning tunneling microscopy and atom probe tomography. The structure and composition of strain-free GaAs/AlGaAs DEQDs was studied in detail for the fundamental understanding of droplet epitaxy growth mechanism. This is needed for further optimization of DEQDs for various optoelectronic applications. The droplet size has shown significant effect on the QD formation. Methods such as flushing technique can be employed to further gain control over QD height. The presence of GaAs intrusions due to local droplet etching of the underlying layer was revealed by X-STM. We presented an X-STM study of MOVPE grown strained InAs/InP DEQDs with atomic resolution. These InAs/InP QDs are potential candidates for quantum communication networks as they emit in low-loss wavelength region of 1.55 μm. The size, shape, and composition of the QDs was determined along with the presence of InAs etch pits in InP. The droplet epitaxy is still being explored to grow QDs with different materials and the structural characterization techniques such as X-STM is a valuable tool to probe the embedded QDs at the atomic level. 

## Data Availability

Not applicable.
